# Fabrication of Bulk Tungsten Microstructure Arrays for Hydrophobic Metallic Surfaces Using Inductively Coupled Plasma Deep Etching

**DOI:** 10.3390/mi15060807

**Published:** 2024-06-20

**Authors:** Zetian Wang, Yanming Xia, Lu Song, Jing Chen, Wei Wang

**Affiliations:** 1 School of Integrated Circuits, Peking University, Beijing 100871, China; zt.wang@pku.edu.cn (Z.W.); l.song@pku.edu.cn (L.S.); 2 National Key Laboratory of Advanced Micro and Nano Manufacture Technology, Beijing 100871, China; 3 Guangzhou National Laboratory, Guangzhou 510005, China; xia_yanming@gzlab.ac.cn; 4 Hicomp MicroTech Co., Ltd., Suzhou 215028, China

**Keywords:** tungsten ICP deep etching, sidewall angle development, metallic hydrophobic surfaces

## Abstract

Hydrophobic surfaces have attracted great attention due to their ability to repel water, and metallic surfaces are particularly significant as they have several benefits, for example they self-clean and do not corrode in marine environments, but also have several applications in the aircraft, building and automobile industries. Tungsten is an ideal material for metallic surfaces due to its remarkable mechanical properties. However, conventional micromachining methods of micro- or nanostructures, including mechanical fabrication and laser and wet etching are incapable of balancing functionality, consistency and cost. Inspired by the etching process of silicon, deep etching of bulk tungsten has been developed to achieve versatile microstructures with the advantages of high efficiency, large scale and low cost. In this article, fabrication methods of tungsten-based hydrophobic surfaces using an ICP deep etching process were proposed. Micro- or hierarchical structure arrays with controllable sidewall profiles were fabricated by optimizing etching parameters, which then exhibited hydrophobicity with contact angles of up to 131.8°.

## 1. Introduction

Hydrophobic surfaces, inspired by aspects of nature, like lotus leaves, butterfly wings and spider silk, result from two main factors, namely micro–nanostructures and materials with low surface energy [1,2,3,4,5]. Air is trapped in the gap between micro–nanostructures, and this prevents water from wetting the bottom surface, which is described by the Cassie–Baxter theory [6,7]. Specifically, metallic hydrophobic surfaces have attracted extensive attention because hydrophobicity can contribute to metals’ functionalities like self-cleaning, anti-corrosion, anti-icing and anti-fogging [8,9,10,11,12,13,14]. Hydrophobic interfaces in metals are typically processed by two main methodologies, i.e., bottom–up approaches and top–down approaches. In bottom–up approaches, a low surface energy layer is deposited on a metallic substrate. Organic coatings are fabricated via spray coating, electrospinning, plasma deposition, etc. [15,16,17,18,19,20,21]. However, coatings’ stability remains challenging in practical applications, limited by both the coating properties and interfaces’ adhesion. Additionally, surface roughness is significant in establishing hydrophobicity. Plasma oxidation and anodic oxidation are most commonly used to fabricate micro- or nanostructures on metallic surfaces, especially aluminum [22,23,24]. Electroplating of nickel microstructure arrays on glass substrates also leads to a superhydrophobic surface [25]. In the top–down approach, laser structuring is widely used due to its no-contact, 3D-patterning ability and wide material-adaptability [26,27]. Laser-induced periodical surface structures (LIPSS), direct laser interference patterning (DLIP) and direct laser writing (DLW) have been reported to modify the surfaces of various metals, including Ti64 and Al2024 [28,29,30,31,32]. Precise mechanical fabrication methods like micro-electrical discharge machining (μEDM) of stainless steel and wire cut electrical discharge machining (WEDM) on aluminum alloy were also used to create hydrophobic or even oleophobic metallic surfaces [33,34]. However, these methods were limited by their relatively low fabrication efficiency, consistency and poor economy.

Inductively coupled plasma reactive ion etching (ICP RIE) is an anisotropic process assisted by excited plasma, and is widely used in the semiconductor industry, benefiting from advantages of high efficiency, large scale and low cost [35,36]. Various methods based on silicon deep etching technology have been reported to achieve hydrophobic surfaces. Silicon micro-needles, pillar or nanostructures with very large negatively tapered profiles can be obtained by modifying the etching strategies, especially gas components [37,38,39]. Additionally, re-entrant and even double re-entrant silicon or silica structures are successfully fabricated by combining anisotropic and isotropic etching [40,41,42,43]. These structures with complicated morphologies show a better water-repelling property than simple micropillar arrays, which strengthens the advantages of the etching process.

Tungsten (W) is widely used in a variety of applications because of its excellent properties, which include a high melting point, high hardness, good chemical inertness, good heat stability and excellent tensile strength [44,45]. These properties make it hard to fabricate, especially through micromachining. Although there are other refractory or anti-corrosion metals with strong characteristics, such as Hf and Ta, tungsten can be relatively easily fabricated by an etching process, since tungsten thin-film etching has been applied in the integrated circuits field as an important interconnect material in ULSI [46,47]. But deep etching of bulk tungsten significantly differs from the etching of thin film due to variations in surface electrical conditions. Inspired by the deep etching process of silicon, efforts have been made in our previous work. Similar to molybdenum-based field emission arrays, fluoric ICP was employed to etch bulk tungsten [48,49,50]. Micro-needle arrays were initially achieved by isotropic etching, and high-aspect-ratio microstructures were micromachined by a mixed reactive plasma of SF_6_ and C_4_F_8_ [51,52,53]. Nevertheless, fabrication of tungsten-based microarrays with varying sidewall angles and nano-textures for water-repelling applications remains unreported.

In this paper, a fabrication method of bulk tungsten microarrays for hydrophobic uses was proposed based on the ICP RIE process. The fabrication of microstructure arrays with various sidewall profiles, including overhang structures, re-entrant structures and hierarchical structures, was achieved through the utilization of the ICP RIE process. Subsequently, contact angles were measured and compared for these aforementioned microstructures, as well as for vertical ones. Notably, the maximum apparent contact angle reached 131.8°. These tungsten-based hydrophobic surfaces suggest promising applicability to self-cleaning, anti-corrosion, anti-icing and anti-fogging.

## 2. Experiment Description

### 2.1. Sample Preparation and Etching Device

**Figure 1:**
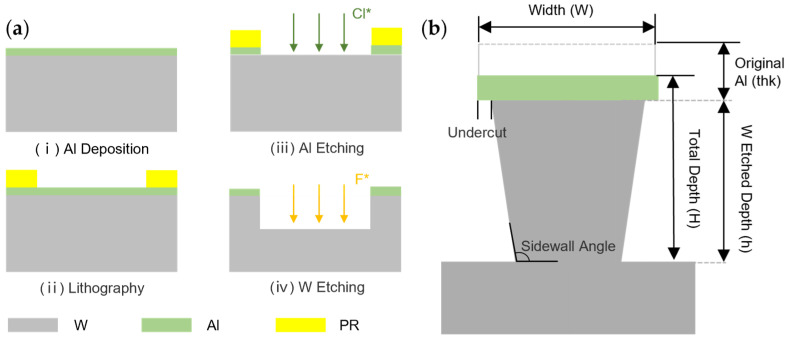
(**a**) Process flow of bulk tungsten ICP etching; (**b**) schematic of critical measurements.

A 4-inch 500 μm thick bulk tungsten wafer was customized by Hicomp Co. Ltd. (Suzhou, China). As shown in Figure 1a, a 4 μm thick aluminum (Al) hard mask was deposited by sputtering (MS150 × 6, FHR, Ottendorf-Okrilla, Germany). Then, photoresist (AZ 4620, 10 μm) was spin coated on the wafer, baked (hotplate, 100 °C, 10 min) and patterned by lithography (MA-6, Suss, Munich, Germany). Then, Al was etched by Cl2, BCl3 in an ICP plasma etcher (SI 500, Sentech, Berlin, Germany) for 13 min, and the process parameter is shown in Table 1. After etching, the wafer was protected by thickly sprayed photoresist (AZ 4620, 25 μm) and divided into multiple 1 cm × 1 cm small samples by laser cutting. Then, the remaining photoresist was removed by acetone and isopropanol.

The tungsten etching was accomplished by a customized ICP plasma etcher (SI 500, Sentech, Berlin, Germany). Programs were edited by Sentech’s professional controlling system software. Process technological parameters, including ICP power (ICP), RF power (RF), pressure (p), temperature (T), gas flow and reaction time (t) were easily set. An antenna of 13.56 MHz, whose ICP power can reach 2500 W on the top surface, was used to excite the plasma and increase the density of reactive particles. A negative RF bias, which can reach 600 W, was used to accelerate ions vertically, and the ions’ typical kinetic energy is several tens of eV. Process gases showered from the top sidewall of the reaction chamber, precisely controlled by corresponding mass flow controllers. And a throttle valve with pumping system was used to control the chamber pressure. The substrate temperature was precisely controlled by a compound thermo-system, including thermocouple, helium backside flow and cycling coolant, with an accuracy of ±0.1 °C. Time determined the reaction time of the whole process according to the etch rate.

### 2.2. Characterized Parameters

After conducting experiments, several characterized parameters needed to be extracted in order to evaluate the etching quality, including etch rate (ER), selectivity (ES), aspect-ratio (AR) and sidewall angle, as shown in Figure 1b. ER represents the speed of the etching process, and it is given by

(1) $$ER=\frac{h}{t}$$

where h is the etched depth of bulk tungsten measured by a surface step profiler (DektakXT, Bruker, Billerica, MA, USA) after removing the Al hard mask by diluted hydrochloric acid; t is the etching time. A consistent ER is crucial for determining the processing time required to achieve the desired etch depth. ES reflects the difference of etching rates between the mask and tungsten, and it is given by

(2) $$ES=\frac{h}{thk+h-H}$$

where thk is the original thickness of the Al mask and is measured before the etching process; H is the total depth of the reserved mask and the etched depth of bulk tungsten, and it is measured before removing the Al mask. A high ES is particularly important in the micromachining of deep structures as it is challenging to etch Al of more than 8 μm thickness. The sidewall angle is the angle between the sidewall and the bottom of the etched structures (termed negative when >90° and vice versa), and undercut is formed by isotropic etching, both of which can be observed directly by scanning the electron scope (SEM, JSM-6390 SEM, JOEL, Tokyo, Japan). And the sidewall angle will be vertical when the etching and passivation are balanced, but negative (>90°) when etching overweighs passivation, and vice versa.

The waste gases of the etching process consist of unused fluorine-based compounds and volatile reaction products, which potentially contribute to the greenhouse effect and are biologically toxic. After being purged from the chamber, they are recycled and treated by local commercial scrubbers and central scrubbers sequentially, which significantly reduces their environmental impact, and they are emitted when the emission standard is met.

### 2.3. Contact Angle Measurement

The wettability of the tungsten microstructure array surface was quantified by using an optical tensiometer (DSA100B, KRÜSS, Hamburg, Germany) with 3 μL droplets of deionized (DI) water in the room temperature and at normal atmospheric pressure. Contact angles between the droplets and the metallic surfaces were recorded by a CCD camera, and the resulting data were analyzed using ImageJ (1.54f).

## 3. Results

### 3.1. Profiles by Different Etching Gas

The DRIE process effectively removed targeted materials through a combination of physical bombardment and chemical reaction, aided by an electromagnetic field. The reaction product was purged from the chamber using gas flow facilitated by vacuum pumps, necessitating a low boiling point. Given that tungsten fluoride exhibits higher volatility compared to its chloride counterpart, fluorine-based etchants were employed for the etching of tungsten membranes. In the case of the high-aspect-ratio etching of bulk tungsten, SF_6_ was primarily selected due to its ability to generate a higher concentration of fluorine radicals within the plasma. The addition of C_4_F_8_ induced the formation of a fluorocarbon passivation layer on the sidewalls during etching, promoting vertical profile formation while enhancing bombardment effects. However, it should be noted that alternative fluorochemicals or their mixtures may yield different etching outcomes.

**Figure 2:**
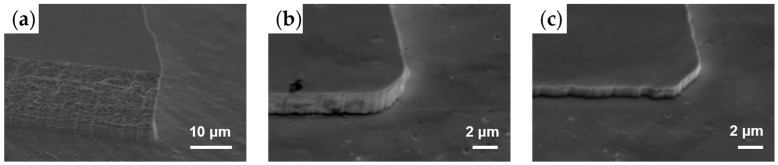
Sidewall profiles after 15 min etching using (**a**) SF_6_; (**b**) CF_4_; (**c**) CHF_3_.

In this study, the etching and passivation effect of CHF_3_ and CF_4_ were investigated. Bulk tungsten was etched by SF_6_, CHF_3_ and CF_4_ under similar conditions, as listed in Table 2. ER of CF_4_ (0.2 μm/min) and CHF_3_ (0.1 μm/min) were notably low compared to that of SF_6_ (2 μm/min). CF_4_ exhibited a faster ER than CHF_3_ due to its higher proportion of fluorine, resulting in a different etching profile in the mixed etching modes. Moreover, the SEM characterization results in Figure 2 revealed that the sidewall steepness achieved by CHF_3_ and CF_4_ was superior to that achieved by SF_6_, with significantly lower sidewall and bottom surface roughness observed. This outcome suggested an extra passivation function of CHF_3_ and CF_4_ due to the polymerization of the fluorocarbon layer. In contrast, C_4_F_8_ demonstrated a poor reactive etching ability. Consequently, diverse etching profiles emerged when processed with the mixture of SF_6_ with CHF_3_ or CF_4_.

### 3.2. Overhang Structures

**Figure 3:**
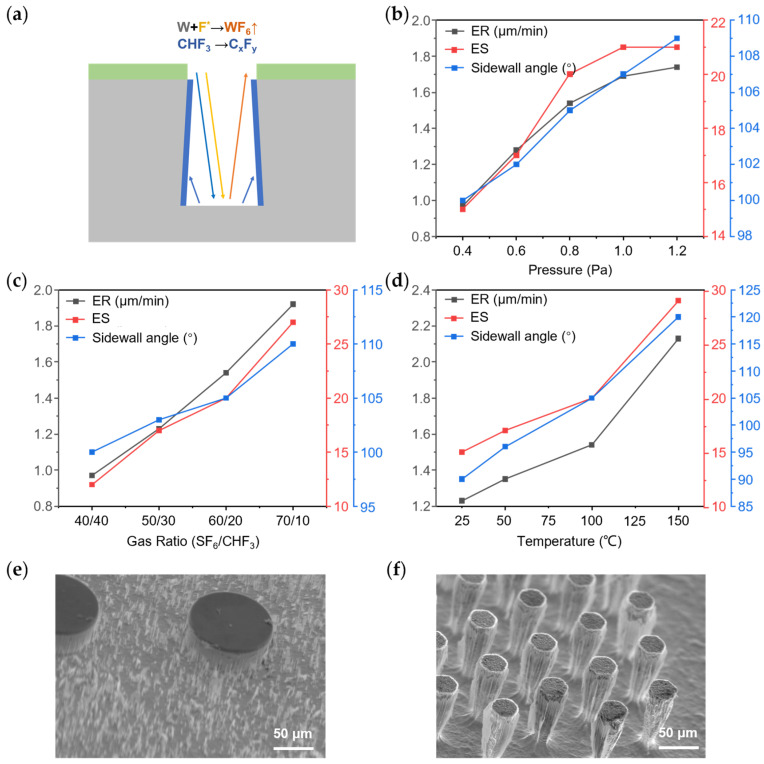
(**a**) Mechanism of etching by mixture of SF_6_ and CHF_3_; ER, ES and sidewall angle dependent on (**b**) pressure, (**c**) gas ratio between SF_6_ and CHF_3_ and (**d**) substrate temperature; (**e**) micro-grassing effect when substrate temperature was set at 25 °C; (**f**) sidewall angle reached 120° when substrate temperature was set at 150 °C.

In the ICP RIE process, CHF_3_ demonstrated both etching and passivation capabilities. An etching profile with a negative sidewall, i.e., overhang structure, was achievable using a mixture of CHF_3_ and SF_6_, as illustrated in Figure 3a. In this process, SF_6_ served as the primary source of fluorine radicals, which reacted with exposed tungsten to produce volatile fluoride. CHF_3_ contributed to passivation on both the sidewall and bottom surfaces. However, the passivation on the bottom surface was easily removed by electric field-accelerated ions, while the carbon-fluoride polymers adhering to the sidewall remained. Since the passivation effect of CHF_3_ was not as effective as that of C_4_F_8_, etching predominated over passivation at the reaction interface, resulting in a negative sidewall angle.

The sidewall angle was able to be adjusted by varying process parameters, including pressure, temperature and gas ratio. In the following single-factor experiments, the original method was set as shown in Table 3. Figure 3b illustrates ER, ES and the sidewall angle results under different pressures ranging from 0.6 to 1.0 Pa. Higher chamber pressure increased the concentration of reactive particles like ions and radicals in plasma, leading to a more ion-assisted chemical etching. This caused a faster ER and larger ES. Chemical etching tends to produce isotropic profiles, so the sidewall angle decreased. The impact of the gas ratio was also examined under a constant total gas flux. Due to the different reaction mechanisms of these gases, the resulting parameters exhibited simple trends, as shown in Figure 3c. Additionally, varying the substrate temperature from 25 °C to 150 °C revealed a similar but more pronounced trend, as depicted in Figure 3d due to the differing temperature dependencies of the etching and passivation reactions. The etching reaction was more sensitive to temperature changes so it outweighed passivation during the increase in temperature, which led to a dominance of etching over passivation as the temperature increased, resulting in a quicker ER, larger ES and smaller sidewall angle. Compared to chamber pressure and gas ratio, temperature changes had a more significant effect on the profiles. At a low temperature of 25 °C, passivation on the bottom surface was insufficiently etched, and the remaining particles acted as micro-masks. Figure 3e shows the emergence of the micro-grassing effect as the etching process continued. Conversely, at a high temperature of 150 °C, the sidewall angle increased to 120° and a thinner sidewall passivation layer formed, as shown in Figure 3f. This led to an increased ER of 2.13 μm/min and an ES of 29, consistent with the analysis above.

**Figure 4:**
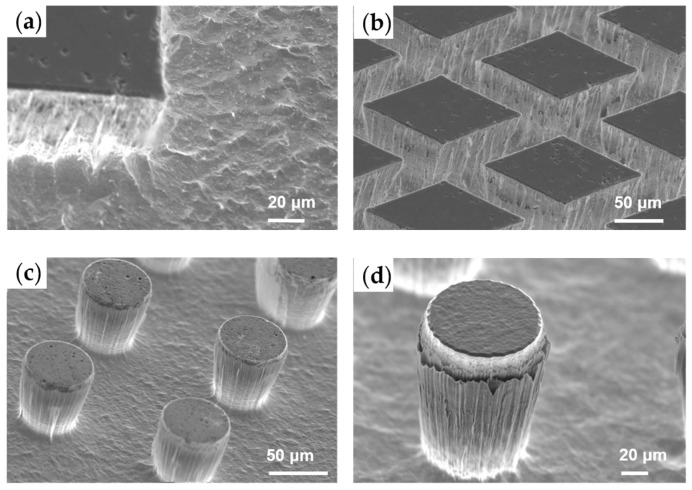
(**a**,**b**) Inverted pyramid microarray; (**c**,**d**) overhang microstructure array for future hydrophobic metallic surface.

The bombardment effect of CHF_3_ was significantly weaker compared to that of C_4_F_8_, resulting in the absence of rounded corners when the mask was patterned in a square configuration, as shown in Figure 4a. Figure 4b illustrated the successful fabrication of an overhang structure array. Figure 4c,d present typical outcomes of microarrays exhibiting a negative sidewall angle, which are intended for the development of future hydrophobic metallic surfaces, using the process parameters detailed in Table 3. The polymerization of the passivation layer on the sidewalls effectively protected the microstructures from further etching, and the bottom surface exhibited low roughness due to the synergistic effects of the etching and passivation processes.

### 3.3. Re-Entrant Structures

**Figure 5:**
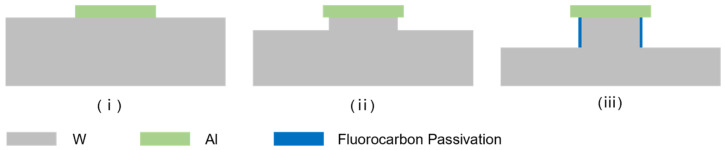
Proceeding schematic of re-entrant microstructure fabrication. (**i**) Al hard mask etching; (**ii**) a mushroom-like structure etched using a mixture of SF_6_ and CF_4_; (**iii**) a re-entrant structure etched using a mixture of SF_6_ and CHF_3_.

Re-entrant structures are known to provide an excellent hydrophobic performance due to their ability to trap air beneath a droplet, thereby preventing liquid from wetting the solid surface. In this study, a two-step etching process was developed to fabricate such structures. As illustrated in Figure 5, the first step involved etching a mushroom-like structure with vertical sidewalls using a mixture of SF_6_ and CF_4_. In the subsequent step, a high-aspect-ratio re-entrant structure was formed using a mixture of SF_6_ and CHF_3_, as per the previously described method.

**Figure 6:**
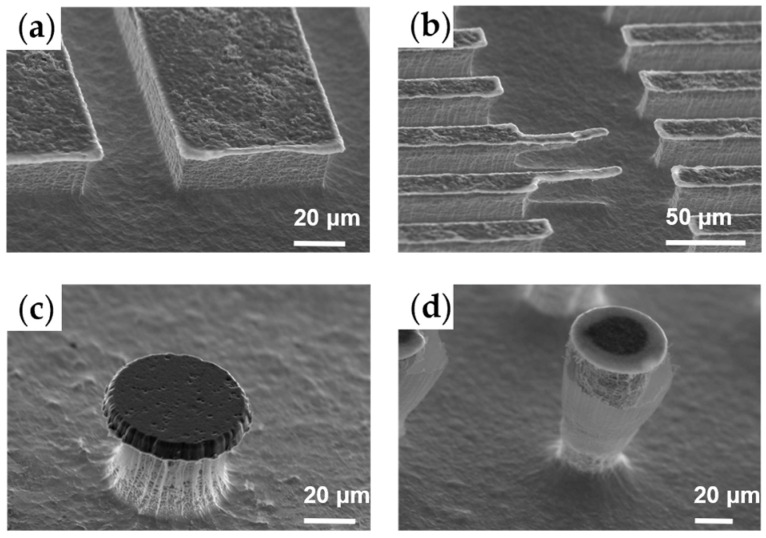
(**a**) Re-entrant structure with vertical sidewall etched by mixture of SF_6_ and CF_4_; (**b**) converging undercuts with the proceeding of process; (**c**) re-entrant structure with aluminum caps after 12 min etching; (**d**) re-entrant microstructure with negative sidewall etched using a two-step method.

The etching of tungsten using a mixture of SF_6_ and CF_4_ was investigated. Due to the inferior passivation performance of CF_4_, the fluorocarbon polymer failed to form a stable sidewall passivation layer. A method similar to that in Table 3, but substituting CF_4_ for CHF_3_ at the same flow rate, was employed. The ER of 1.65 μm/min and ES of approximately 25 were both higher than those achieved with CHF_3_ due to the aforementioned reasons. The etching results, as shown in Figure 6a, indicated a significant undercut beneath the aluminum hard mask. However, the sidewall angle was nearly 90°, in contrast to the isotropic profiles observed with SF_6_. Microstructures with smaller critical dimensions tended to disappear when undercuts from both sides converged, as shown in Figure 6b, which is unacceptable for the fabrication of high-aspect-ratio micropillar arrays. Therefore, after 12 min of etching with the SF_6_/CF_4_ mixture, re-entrant structures with aluminum caps appeared, as shown in Figure 6c. The process was then switched to the method using SF_6_ and CHF_3_ to protect the sidewalls from further etching. Figure 6d shows the resulting etching profile of a high-aspect-ratio re-entrant microarray. A passivation layer formed during the second step of the process, and the overall profile exhibited a negative sidewall angle, which further contributed to its hydrophobic performance.

### 3.4. Hierarchical Structures

Photoresist is commonly employed as a mask material in the DRIE process of silicon. However, for bulk tungsten etching, an aluminum hard mask was selected and developed due to the substantial ion bombardment induced by higher RF power. This choice, however, increased process complexity, economic costs and time requirements. By introducing a thick photoresist, hierarchical structure arrays were achieved.

An optimized WIDE (W ICP deep etching) method from our previous work was selected to etch bulk tungsten, using a 50 μm thick AZ50XT photoresist mask patterned through lithography [51]. Briefly, the gas mixture consisted of SF_6_ (40 sccm), O_2_ (10 sccm) and C_4_F_8_ (30 sccm), and this was excited under specific conditions of pressure (0.8 Pa), temperature (150 °C), ICP power (1500 W) and RF power (150 W). In this method, SF_6_ served as the main source of fluoride radicals, while C_4_F_8_ decomposed and polymerized to form fluorocarbon passivation layers. The addition of O_2_ enhanced both the ER and ES. This method enabled the achievement of nearly vertical sidewall profiles using an aluminum hard mask. However, the bombardment effect due to high RF power and the relatively large molecular mass of C_4_F_8_ led to a noticeable chamfering of rectangular patterns, even with the use of a hard mask.

**Figure 7:**
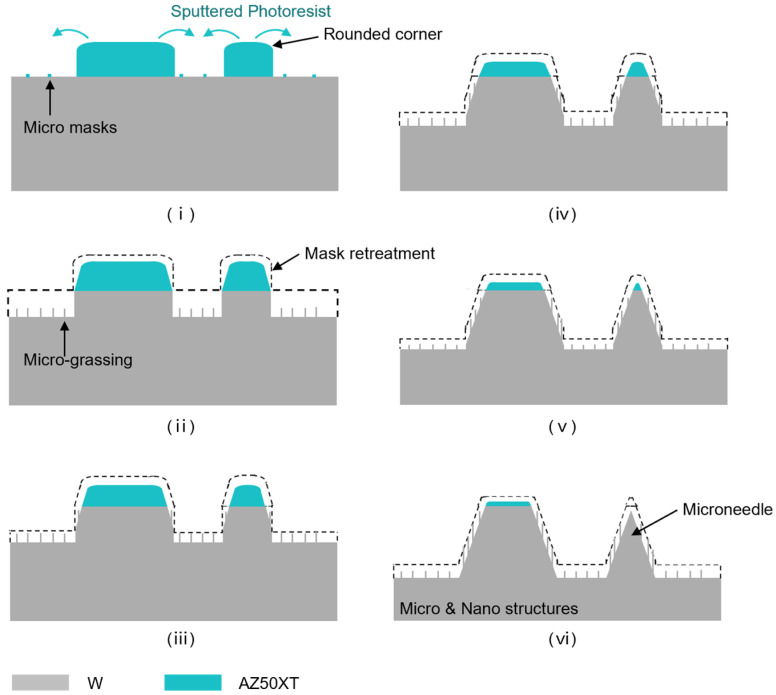
Proceeding schematic of positive-sidewall-angle etching using photoresist as soft mask. (**i**) profiles of PR and sputtered PR; (**ii**) mask retreatment effect and micro-grassing effect; (**iii**–**vi**) proceeding profiles of hierarchical structures.

Additionally, AZ50XT exhibited rounded corners after development and baking, leading to a thickness gradient near the edge of photoresist. Combined with the significant bombardment effect of C_4_F_8_ on the relatively soft AZ50XT, metallic array with a positive sidewall angle could be achieved. And this bombardment effect could be further enhanced by the addiction of 10 sccm argon in the gas flow. As shown in Figure 7, vertical profiles could be obtained at the beginning of the process. However, the mask’s edge would be removed more rapidly due to continuous bombardment, which was facilitated by the concentrated electromagnetic field. As the process continued, the tungsten beneath the edge became exposed to the etchant, a phenomenon referred to as the mask-retreatment effect. This sequential exposure of tungsten from edge to center resulted in a cross-sectional shape with a positive sidewall angle, even producing profiles resembling micro-needles.

**Figure 8:**
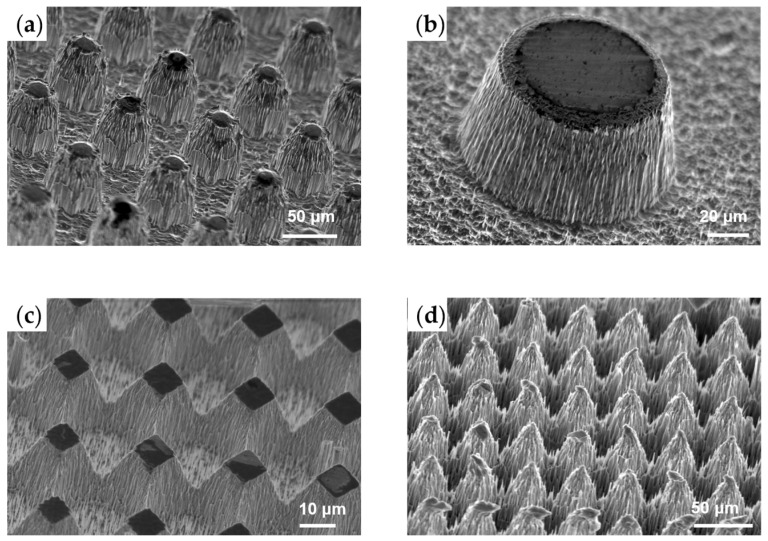
SEM pictures of hierarchical structures. (**a**) Microarray with round pattern; (**b**) nanostructures on the sidewall and bottom due to micro-grassing effect; (**c**) micro-pyramid array with square pattern; (**d**) micro-needle array evolved from (**c**).

Additionally, photoresist particles were sputtered by the bombardment of ions onto the surface of the mask, sidewalls and bottom due to the incomplete reaction of the mask. These particles adhered to the sidewalls and bottom, acting as micro-masks in subsequent etching stages. And micro-masks led to the formation of complex nano-scale secondary structures through a similar mechanism as previously described. These hydrophobic fluorocarbon micro-grassing nanostructures contributed to a larger contact angle, even though microarrays with positive sidewalls theoretically did not exhibit superior performance. Figure 8a showed a typical experimental result of microarrays with positive sidewall angle. A sidewall angle of approximately 70° was achieved using this method, with remarkable nanostructures on the sidewall and bottom, as shown in Figure 8b. The square mask pattern resulted in a pyramid array, with the units connected due to continuous passivation by C_4_F_8_, as shown in Figure 8c. A micro-needle array could eventually evolve when the mask was completely removed by the etchant. The determined ER was about 0.5 μm/min, which is lower than that achieved using the Al hard mask because of the non-negligible micro-grassing effect, and the ES was approximately 1.4, remarkably lower than Al.

### 3.5. Contact Angle

**Figure 9:**
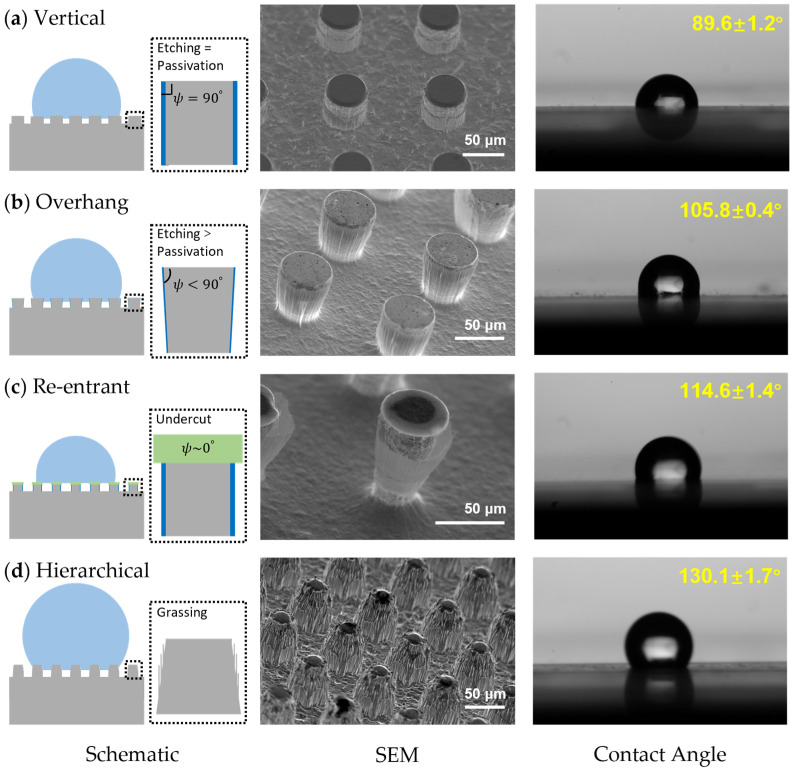
Schematic, SEM characterization and contact angles measurement of (**a**) micropillar array with vertical sidewall angle, (**b**) overhang microstructure array, (**c**) re-entrant micropillar array and (**d**) hierarchical micropillar array.

Contact angles (CA) of four microstructure arrays were characterized, as shown in Figure 9. The etching process of these four microstructure arrays started with the same square mask pattern, with edge and gap lengths of 50 μm each, and an etching depth of 100 μm achieved by adjusting the process time. Using the WIDE method reported in our previous work, a micropillar array with vertical sidewall angle was fabricated [51]. The CA of water on this micropillar array was nearly 90°, compared to the intrinsic CA of metal tungsten, which is around 70°. This transformation can be explained by the Cassie–Baxter model:

(3) $$\cos\theta_{e}^{C}=-1+f_{s}\left(1+\cos\theta_{e}\right)$$

where $\theta_{e}$, $\theta_{e}^{C}$ and $f_{s}$ represent the intrinsic CA, the apparent CA on the composite surface, and the solid-area fraction of the surface, respectively. ψ is the local geometric angle of the texture. Due to tungsten’s hydrophilicity, ψ is larger than $\theta_{e}$, promoting water penetration into the solid texture until a balance state is reached, which resulted in a measured CA smaller than the CA calculated by Equation (3). Furthermore, ψ became smaller in the micropillar with a negative sidewall angle, which is also known as an overhang microstructure, as shown in Figure 9b, leading to a larger CA of 106°. This increase in CA can be attributed to the enhanced ability of the overhang structures to trap air beneath the water droplets, reducing the contact between the water and the tungsten surface, thus increasing the overall hydrophobicity. In addition, the passivation layer deposited on the sidewall, consisting of fluorocarbon polymer, also repels water from further wetting since CF_x_ groups exhibit low surface energies [54].

In comparison, the re-entrant microstructure can be considered an extreme case of the overhang structure, and it exhibited an even higher CA. Water is pinned at the undercut profiles, resisting further penetration into the gaps between the microstructures. The apparent contact angle (CA) of the re-entrant microstructure array was 115°, surpassing the overhang array. However, the undercut profiles were formed due to the isotropic etching of tungsten using an SF_6_/CF_4_ mixture, necessitating the retention of the aluminum hard mask. This requirement can degrade the mechanical performance of the metallic surfaces. Conversely, hierarchical structures, resulting from the micro-grassing and mask-retreatment effects, demonstrated the most excellent water-repelling properties, with a CA as high as 131.8°. Although the sidewall angle of this hierarchical structure was positive, leading to a larger ψ, numerous tiny air pockets formed between the gaps of the nanostructures, counteracting the surface tension. In addition, due to much closer spacing, the condensate is nucleated on top of the nano-textures since these nano gaps make it more difficult for the vapor to be diffused. This phenomenon contributes to the reduction of the condensate micro-droplets’ pinning force, which leads to the better depinning properties of the hierarchical structures. In summary, both micro- and hierarchical structures can be used to create tungsten-based hydrophobic surfaces. The wettability can be further enhanced by optimizing the pattern design and process parameters. Compared to traditional hydrophobic coatings, metallic surfaces fabricated by this method show a better mechanic strength, retaining maximum long-term reliability under extreme temperature and pressure conditions.

## 4. Conclusions

The fabrication process of various tungsten-based hydrophobic surfaces was developed using ICP deep etching, and the apparent contact angles could be increased by up to 131.8°. Micropillar arrays with overhang or re-entrant profiles were achieved through the use of gas mixtures of SF_6_ and different fluorocarbons. The etch rate, selectivity and sidewall angle were effectively modulated by adjusting key process parameters, such as pressure, temperature and gas ratio. Furthermore, a hierarchical microstructure array based on mask-retreatment and micro-grassing effect was fabricated employing photoresist as the etching mask. The wettability of these fabricated structures, including vertical pillar, overhang, re-entrant and hierarchical microarrays was systematically characterized. The excellent mechanical properties of tungsten, combined with the enhanced hydrophobicity of these micromachined surfaces, suggest significant potential for applications in self-cleaning and anti-corrosion technologies.

## Figures and Tables

**Table 1:** Process parameters used in Al etching.

ICP	RF	P	Cl_2_	BCl_3_	T	t
250 W	150 W	0.3 Pa	30 sccm	20 sccm	0 °C	15 min

**Table 2:** Process parameters used in etching function comparison among SF_6_, CHF_3_ and CF_4_.

ICP	RF	P	Gas Flow	T	t
1500 W	150 W	0.6 Pa	40 sccm	150 °C	15 min

**Table 3:** Process parameters used in etching by mixture of SF_6_ and CHF_3_.

ICP	RF	P	SF_6_	CHF_3_	T
1500 W	150 W	0.8 Pa	60 sccm	20 sccm	100 °C

## Data Availability

The original contributions presented in the study are included in the article, further inquiries can be directed to the corresponding authors.
